# A Wolf in Sheep’s Clothing: Collision of Melanoma and Keratoacanthoma

**DOI:** 10.3390/dermatopathology8030030

**Published:** 2021-07-04

**Authors:** Matthias Walther, Sandra Falkvoll, Sebastian Leibl

**Affiliations:** 1 Skinmed—Clinic for Dermatology, 5000 Aarau, Switzerland; sfalkvoll@skinmed.ch; 2 Skinpath—Histopathology, 5600 Lenzburg, Switzerland; sleibl@skinpath.ch

**Keywords:** melanoma, squamous cell carcinoma, collision tumor, oncology, dermatopathology

## Abstract

Collision tumors consisting of melanoma and squamous cell carcinoma are very rare. We present the case of a deceptive hyperkeratotic nodule on the forearm of a 72-year-old woman, which clinically appeared to be a squamous cell carcinoma, keratoacanthoma type. Histological examination surprisingly revealed a coexisting epithelioid melanoma. Thus, this case report shows the importance of an early histopathological and immunohistochemical workup to prevent unnecessary diagnostic and therapeutic delay with negative effects on prognosis.

## 1. Case Presentation

**Figure 1:**
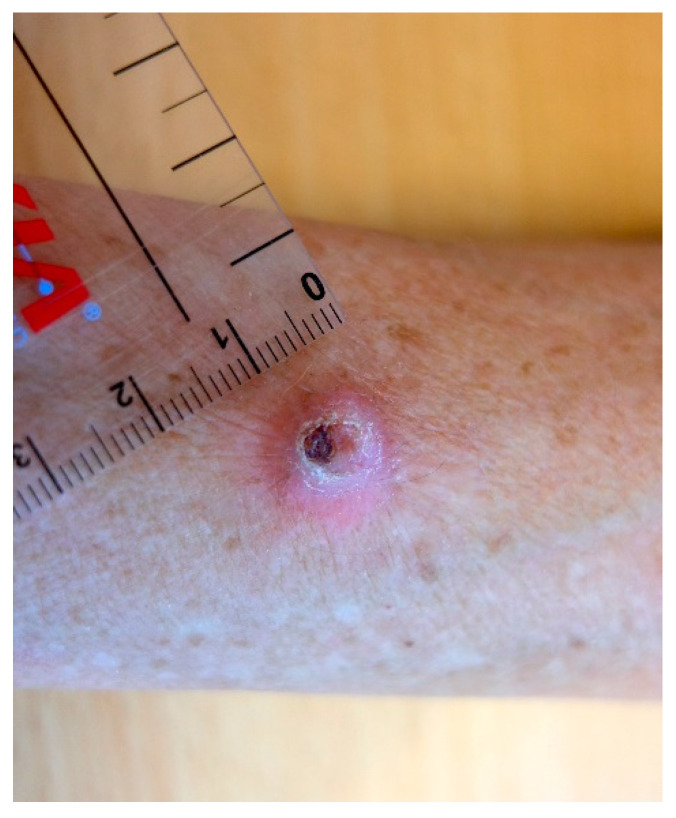
Macroscopy, hyperkeratotic nodule on forearm.

A 72-year-old female patient (Fitzpatrick skin phototype 2, no family history of melanoma) presented with a slightly red, hyperkeratotic, and crusty nodule on her right forearm (Figure 1), which had quickly developed during the last few months in sun-damaged skin with multiple solar lentigines. Clinically, the lesion was suspicious for squamous cell carcinoma/keratoacanthoma. The medical history of the patient was unremarkable except for arterial hypertension and some actinic keratoses in the past.

**Figure 2:**
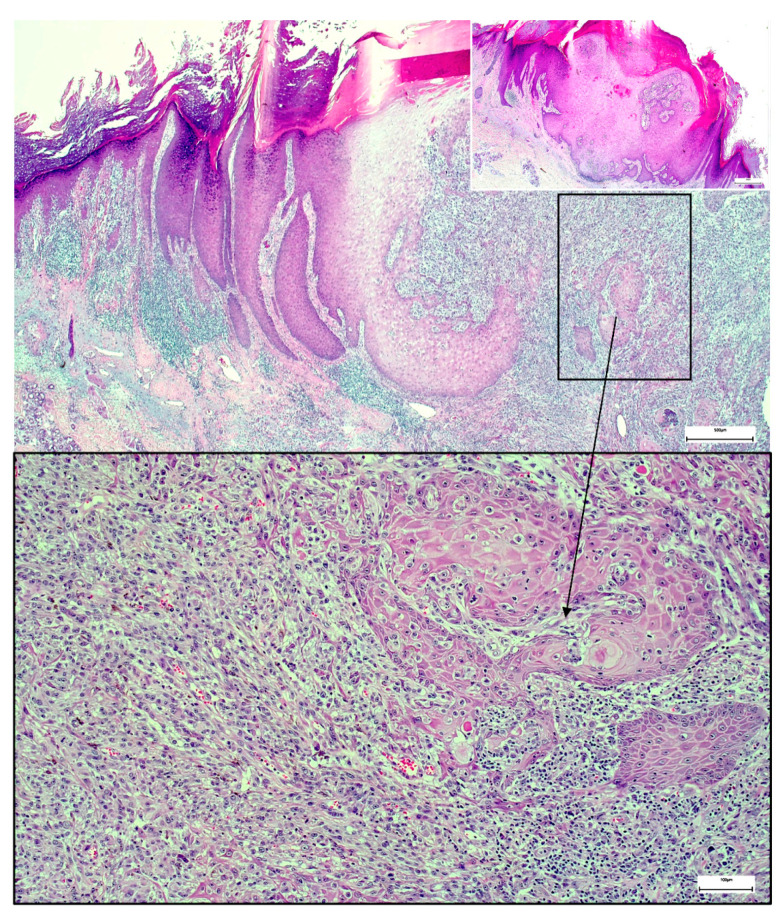
Collision tumor consisting of keratoacanthoma and melanoma, Hematoxylin and Eosin stain (H&E). The small insert on the upper right side shows the edge of the keratoacanthoma with characteristic architecture and large pale keratinocytes.

Three weeks after the first consultation, the nodule was excised with a safety margin of about 3 mm. Surprisingly, histology revealed a collision tumor consisting of keratoacanthoma and melanoma with a Breslow thickness of 4.6 mm (Figure 2). Consequently, the patient was referred to a hospital specialized in melanoma for further diagnostics and therapy (PET-CT scan and evaluation of sentinel lymph node biopsy). Unfortunately, the PET-CT scan showed pulmonary metastases, which were histologically confirmed by fine-needle aspiration. Neither BRAF nor NRAS mutations were found in the tumor genome analysis. The patient is currently treated with anti-PD1 immunotherapy. The first PET-CT staging after 3 months showed a therapy response (smaller pulmonary nodules and no new lesions) under ongoing therapy.

## 2. Discussion

Collision tumors consisting of melanoma and squamous cell carcinoma (called melanocarcinoma in the literature) are very rare, and their biological potential is unknown [1]. Various theories try to explain their occurrence: as most collision tumors occur on sun-damaged and aged skin, the cancerization theory favors the development of two intermingled neoplasms from two phenotypically distinct clones [2,3]. Paracrine stimulation as a reason for the development of two different malignancies at the same site is discussed, but their simultaneous appearance could also just be a coincidence, as stated by the tumor divergent theory [3,4,5]. In this theory, the neoplastic cells arise independently before colliding [3]. On the other hand, the so-called tumor convergent theory explains the development of collision tumors through multipotent stem cells undergoing dual differentiation [3,6,7].

Although no theory has to date been confirmed [3], collision tumors can be histologically subdivided into an adjacent or intermingled growth pattern [8]. Some authors have described melanomas within seborrheic keratoses or in combination with basal cell carcinomas [5,7,9]. Kochoumian et al. also report a case of metastatic melanoma in collision with squamous cell carcinoma [1]. The squamo-melanocytic tumor was described as a mostly dermal nodule with intimately admixed melanoma and clear-cut squamous cell carcinoma [10].

**Figure 3:**
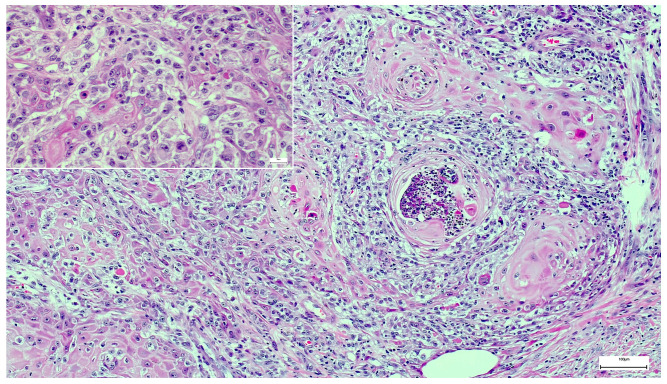
Detail of the invasion front: Large pale keratinocytes and epithelial nests with micro abscesses in a background of spindle-shaped melanoma cells (H&E stain).

**Figure 4:**
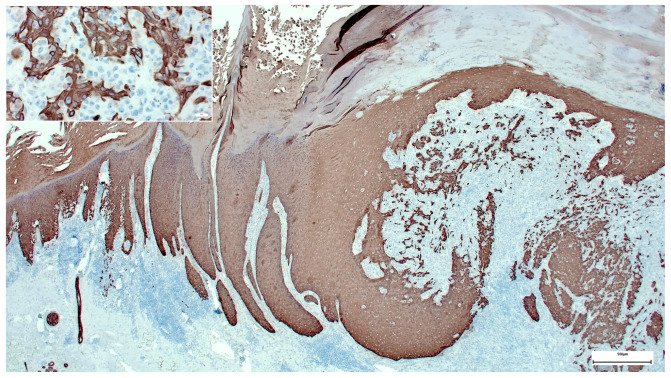
High molecular weight cytokeratin (clone 34βE12) stain highlighting the epithelial component of the collision tumor.

**Figure 5:**
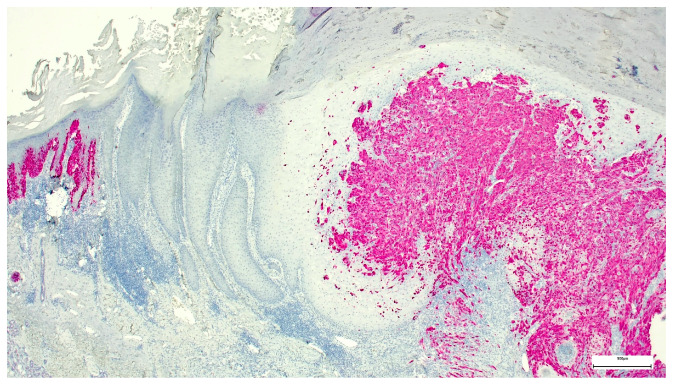
Melan-A immunohistochemical stain highlighting the melanoma component of the collision tumor.

In our case, the overall histological picture showed an invaginating squamous proliferation consistent with keratoacanthoma/well-differentiated squamous cell carcinoma, keratoacanthoma type (Figure 2). This squamous lesion exhibited characteristic lipping of the edges, large pale keratinocytes, and micro abscesses in epithelial nests at the bottom (Figure 2 and Figure 3). However, a conspicuous diffuse spindle cell proliferation expressing high molecular weight cytokeratin (clone 34βE12) with interspersed islands of more differentiated atypical squamous epithelium suggested transition into poorly/undifferentiated squamous cell carcinoma (Figure 4). Further examination of the epidermis adjacent to the tumor revealed a junctional spindle cell proliferation suspicious of melanoma in situ and a few solar lentigines further away from the tumor. Subsequent immunohistochemical analysis not only confirmed the melanoma in situ but also showed positive melanocytic markers (Melan-A, HMB45, SOX10, S100) in the invasive spindle cell component of the tumor (Figure 5), whereas epithelial markers (AE1/3, 34βE12) were negative.

## 3. Conclusions

Our report emphasizes the importance of timely tumor excision and thorough histological examination, even in lesions that are clinically unsuspicious and appear to follow an indolent course.
